# Interleukin-1 primes human mesenchymal stem cells towards an anti-inflammatory and pro-trophic phenotype in vitro

**DOI:** 10.1186/s13287-017-0531-4

**Published:** 2017-04-17

**Authors:** Elena Redondo-Castro, Catriona Cunningham, Jonjo Miller, Licia Martuscelli, Sarah Aoulad-Ali, Nancy J. Rothwell, Cay M. Kielty, Stuart M. Allan, Emmanuel Pinteaux

**Affiliations:** 1 Faculty of Biology, Medicine and Health, University of Manchester, Manchester, UK; 2 Wellcome Trust Centre for Cell-Matrix Research, Faculty of Biology, Medicine and Health, University of Manchester, Manchester, UK

**Keywords:** Stroke, Human mesenchymal stem cells, Cytokines, Priming, Bone marrow-derived stromal cells, Interleukin-1

## Abstract

**Background:**

Inflammation is a key contributor to central nervous system (CNS) injury such as stroke, and is a major target for therapeutic intervention. Effective treatments for CNS injuries are limited and applicable to only a minority of patients. Stem cell-based therapies are increasingly considered for the treatment of CNS disease, because they can be used as in-situ regulators of inflammation, and improve tissue repair and recovery. One promising option is the use of bone marrow-derived mesenchymal stem cells (MSCs), which can secrete anti-inflammatory and trophic factors, can migrate towards inflamed and injured sites or can be implanted locally. Here we tested the hypothesis that pre-treatment with inflammatory cytokines can prime MSCs towards an anti-inflammatory and pro-trophic phenotype in vitro.

**Methods:**

Human MSCs from three different donors were cultured in vitro and treated with inflammatory mediators as follows: interleukin (IL)-1α, IL-1β, tumour necrosis factor alpha (TNF-α) or interferon-γ. After 24 h of treatment, cell supernatants were analysed by ELISA for expression of granulocyte-colony stimulating factor (G-CSF), IL-10, brain-derived neurotrophic factor (BDNF), nerve growth factor (NGF), IL-1 receptor antagonist (IL-1Ra) and vascular endothelial growth factor (VEGF). To confirm the anti-inflammatory potential of MSCs, immortalised mouse microglial BV2 cells were treated with bacterial lipopolysaccharide (LPS) and exposed to conditioned media (CM) of naïve or IL-1-primed MSCs, and levels of secreted microglial-derived inflammatory mediators including TNF-α, IL-10, G-CSF and IL-6 were measured by ELISA.

**Results:**

Unstimulated MSCs constitutively expressed anti-inflammatory cytokines and trophic factors (IL-10, VEGF, BDNF, G-CSF, NGF and IL-1Ra). MSCs primed with IL-1α or IL-1β showed increased secretion of G-CSF, which was blocked by IL-1Ra. Furthermore, LPS-treated BV2 cells secreted less inflammatory and apoptotic markers, and showed increased secretion of the anti-inflammatory IL-10 in response to treatment with CM of IL-1-primed MSCs compared with CM of unprimed MSCs.

**Conclusions:**

Our results demonstrate that priming MSCs with IL-1 increases expression of trophic factor G-CSF through an IL-1 receptor type 1 (IL-1R1) mechanism, and induces a reduction in the secretion of inflammatory mediators in LPS-activated microglial cells. The results therefore support the potential use of preconditioning treatments of stem cells in future therapies.

**Electronic supplementary material:**

The online version of this article (doi:10.1186/s13287-017-0531-4) contains supplementary material, which is available to authorized users.

## Background

Stem cells are undifferentiated cells found in many adult tissues, the function of which is renewal of damaged tissues during ageing or after disease and injuries. Because of their regenerative properties, these cells are being increasingly considered as new therapeutic agents for the treatment of central nervous system (CNS) diseases. Mesenchymal stem (or stromal) cells (MSCs) are one type of multipotent stem cells that can be isolated and cultured from several tissues [1, 2] and differentiated into several cell lineages [3, 4]. MSCs sense signals from tumours [5] and injured, inflamed or ischemic tissues [6], migrate towards those sites and are even able to cross the blood–brain barrier [7]. Once infiltrated into the brain, MSCs produce an array of mediators such as cytokines and growth factors [8, 9] that promote tissue repair mainly by activating endogenous repair mechanisms [10, 11], and by acting as temporal immune-suppressants [1, 6]. These properties make MSCs ideal candidates for cell-based therapies, particularly for the treatment of CNS disorders such as stroke, Huntington’s disease, amyotrophic lateral sclerosis or Parkinson’s disease [12]. The safety and efficacy of MSCs have already been demonstrated mostly in pre-clinical models of ischaemic stroke [2, 9, 13], intracerebral haemorrhage [14], amyotrophic lateral sclerosis [15] and Alzheimer’s disease [16]. However, the precise mechanisms by which MSCs exert beneficial effects remain elusive [17].

A process implicated in the pathogenesis of CNS disorders is inflammation, a key host defence response to infection and injury. Inflammation is known to contribute to neuronal injury [18], but is also implicated in repair mechanisms in the brain [19]. Achieving the right balance between the damaging and reparative role of inflammation is considered a major target for therapeutic intervention [20]. Although some anti-inflammatory treatments are in clinical trial for acute brain injury, current effective treatments are limited [21, 22]. Finding new therapies with longer time windows and wider therapeutic effects has become a priority. Inflammation in the brain is critically regulated by inflammatory cytokines such as interleukin (IL)-1 and tumour necrosis factor alpha (TNF-α) that are expressed by, and act on, microglia, astrocytes and endothelial cells [23]. Studies using MSCs delivered to the brain have been carried out [24, 25], but no studies have yet assessed the effect of the inflammatory environment on the secretory profile of these locally delivered MSCs.

The MSC secretome can be modulated to boost the beneficial actions of these cells, so that they can respond even more effectively to inflammatory conditions. One way to increase this potential is through priming or preconditioning. Inflammatory priming occurs when a mild (or sub-lethal) inflammatory event induces cellular changes that drive cells towards a more anti-inflammatory phenotype, which can eventually lead to a more effective response against future lethal or severe inflammatory events. Different preconditioning treatments have been tested in MSCs in order to induce selected phenotypes [26, 27], but the effect of specific cytokines known to regulate inflammation in the brain or the secretory profile of MSCs has not been widely studied. Here, we tested the hypothesis that priming with inflammatory stimuli would modify the secretome of MSCs towards an anti-inflammatory and trophic phenotype [28]. We show for the first time that MSCs express high constitutive levels of key anti-inflammatory and trophic factors, and that priming with IL-1 triggers secretion of the trophic factor granulocyte-colony stimulating factor (G-CSF), an effect that was only observed in response to IL-1. The addition of IL-1-primed MSC conditioned media (CM) to inflamed microglial cells caused a reduction in the secretion of inflammation markers (IL-6, G-CSF and TNF-α), and an increase in the microglial-derived anti-inflammatory mediator cytokine IL-10. These results highlight the ability of MSCs to orchestrate other cells to induce a more effective anti-inflammatory response, demonstrating the potential use of priming inflammatory treatments to enhance the beneficial actions of MSCs for future stroke therapies.

## Methods

### Human MSCs

Human bone marrow-derived MSCs were purchased from Lonza (UK) and 3H Biomedical (Sweden). Three different donors were used in this study: donor 1 (Lonza, 38 years old, male), donor 2 (Lonza, 21 years old, female) and donor 3 (3H Biomedical, 22 weeks old, fetal). Culture flasks (Corning, UK) were coated with 0.1% gelatin in PBS, overnight at 37 °C, and washed with PBS. MSCs were subsequently cultured in MesenPRO RS medium (Invitrogen, UK) supplemented with 1% penicillin/streptomycin and 2 mM glutamine. The medium was changed every 4–5 days until cells reached 70–80% confluency. Cells were then detached with 0.5% trypsin–EDTA (Sigma-Aldrich, UK), counted and split into different tissue culture flasks and further cultured as already described. Cells used for experiments were obtained from culture passages 4–6 and were seeded in gelatin-coated plates at 13,000 cells/cm^2^, 24 h prior to treatment.

### Differentiation of MSCs and flow cytometry

Cells obtained from different donors were tested for their ability to differentiate into osteocytes or adipocytes using commercial kits (Millipore, UK), according to the manufacturer’s instructions. MSCs were further characterised phenotypically by multicolour flow cytometry using the BD Stemflow™ hMSC Analysis Kit (BD Biosciences, UK) on a FACSVerse flow cytometer (BD Biosciences, UK). The surface markers used for the phenotypic characterisation of MSCs were CD73, CD90, CD105, CD34, CD11b, CD19, CD45 and HLA-DR, as stated by the International Society for Cellular Therapy [29].

### Microglial cells (BV2 cells)

BV2 cells (ATCC, UK), an immortalised murine microglial cell line, were cultured in RPMI-1640 medium (Sigma-Aldrich, UK) supplemented with 10% FBS (Gibco, UK) and 1% penicillin/streptomycin (Sigma-Aldrich, UK) until 70–80% confluent. Cells were detached with trypsin–EDTA (Sigma-Aldrich, UK), counted and seeded at a density of 13,000 cells/cm^2^.

### Cell treatments

#### MSC priming

MSC cultures were treated with recombinant human IL-1α, IL-1β, TNF-α or interferon gamma (IFN-γ) (all from R&D Systems UK) at a final, concentration of 1, 10, 50 or 100 ng/ml. After 24 h of treatment, culture supernatants were collected and analysed for the presence of several cytokines (see “Enzyme-linked immunosorbent assay”).

#### Blocking IL-1 receptor antagonist experiments

IL-1 receptor antagonist (IL-1Ra) (200 μg/ml, Kineret®; Biovitrum, Sweden) was added to MSCs. After 10 min, priming treatments were added normally, without washing (final concentration of 100 μg/ml for IL-1Ra and 10 ng/ml for inflammatory cytokines). After 24 h, supernatants were collected and analysed.

#### Conditioned medium treatment of BV2 cells

**Fig. 1:**
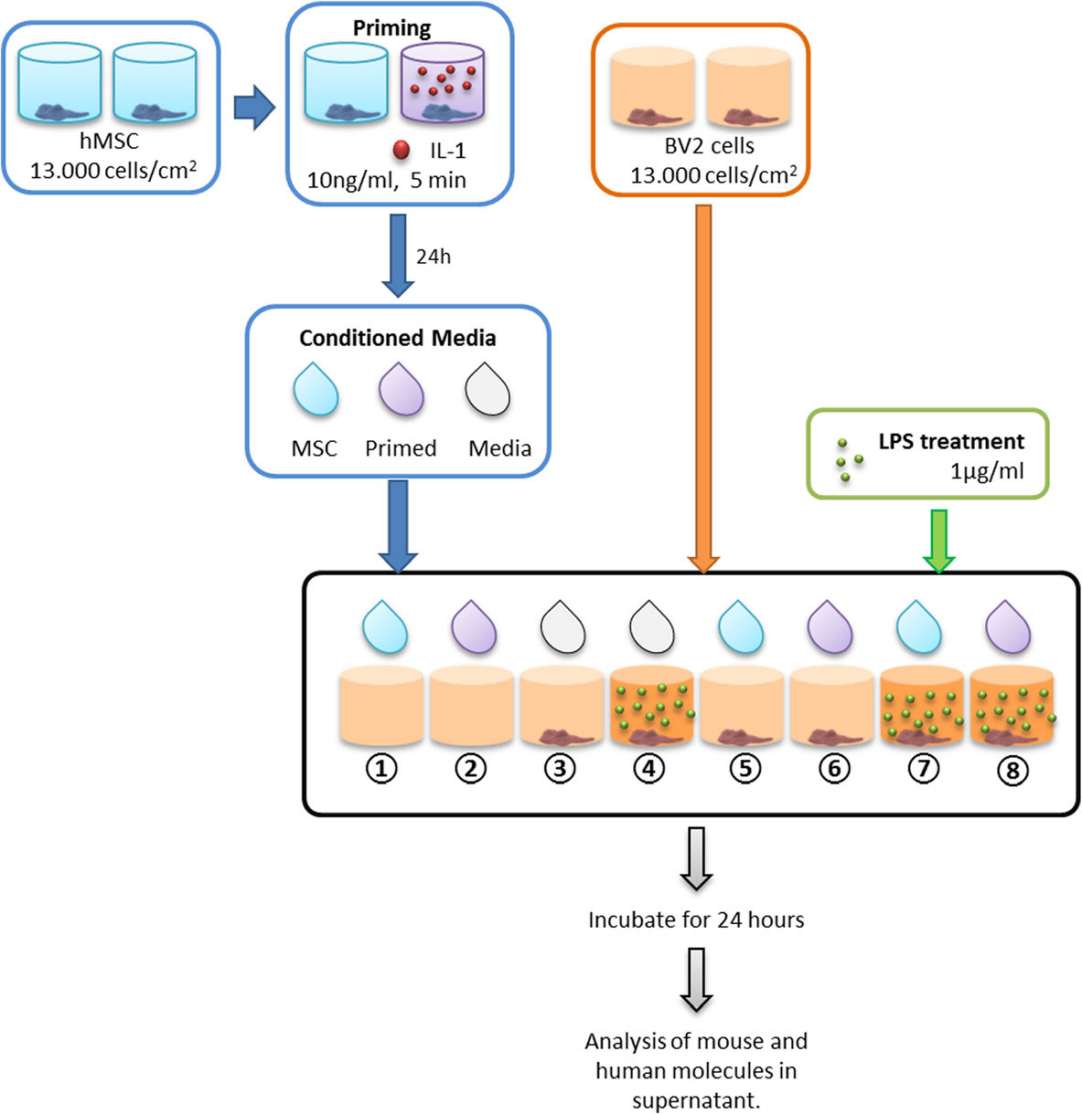
Experimental protocol of MSC-CM treatment on BV2 cells. MSCs were primed with IL-1α, and the CM used on BV2 cells (treated or not with LPS). Summary of experimental conditions: *1*, MSC-CM; *2*, primed MSC-CM; *3*, untreated BV2; *4*, BV2 treated with LPS; *5*, BV2 treated with MSC-CM; *6*, BV2 treated with primed MSC-CM; *7*, BV2 treated with LPS and MSC-CM; *8*, BV2 treated with LPS and primed MSC-CM. *hMSC* human mesenchymal stem/stromal cell, *IL* interleukin, *LPS* lipopolysaccharide

MSCs were seeded at a density of 13,000 cells/cm^2^ in 24-well plates (Corning, UK). Once attached, cells were primed with 10 ng/ml of human recombinant IL-1α (R&D Systems, UK) for 5 min. MSCs were then washed twice with PBS, and fresh MesenPRO medium was added. After 24 h of incubation, CM were collected. BV2 cells were simultaneously treated with CM and 1 μg/ml lipopolysaccharide (LPS) from *E. coli* 0127:B8 (Sigma-Aldrich, UK). Supernatants were collected at 24 h—see detailed experimental protocol (Fig. 1).

#### Blocking human G-CSF experiments

CM from MSCs (primed and unprimed) were incubated in plates previously coated with a neutralising antibody against human G-CSF (R&D systems, UK). After 2 h of incubation at RT and sterile conditions, CM were collected and added to BV2 cells as already described, and cytokines were measured.

#### Lactate dehydrogenase assay

Cell death and proliferation of BV2 cells after cytokine treatments were analysed using a lactate dehydrogenase (LDH) assay kit (Promega, UK) according to the manufacturer’s instructions. In brief, to assess cell death, supernatants were collected, LDH was measured and optical densities were normalised to 100% cell death control. To assess proliferation, all cells were lysed and measured LDH concentrations were compared with control values (untreated BV2 cells). An increase in LDH measurements was interpreted as an increase in cell death or proliferation (respectively).

### Enzyme-linked immunosorbent assay

Levels of human IL-10, brain-derived neurotrophic factor (BDNF), nerve growth factor (NGF), vascular endothelial growth factor (VEGF), TNF-α and G-CSF in culture media from MSCs were quantified by ELISA using DuoSet® kits (R&D Systems, UK) according to the manufacturer’s instructions. Human IL-1Ra levels were measured using an ELISA kit from Peprotech (UK) combined with external standards prepared using recombinant human IL-1Ra (National Institute for Biological Standards and Controls (NIBSC), UK). Quantification limits in human ELISAs were 10 pg/ml for IL-1Ra, 15 pg/ml for G-CSF, NGF, TNF-α and VEFG, and 25 pg/ml for BDNF and IL-10. ELISA kits for mouse IL-6, TNF-α, IL-10 and G-CSF (all quantification limits ~30 pg/ml) were purchased from R&D Systems and used following the manufacturer’s instructions. For each assay, samples were diluted as needed and protein levels were calculated against a four-parameter logistic (4-PL) curve fit. All values are expressed as mean ± standard error of the mean (SEM).

### Statistical analysis

In each experiment, a minimum of four independent cultures were included. Graphs, 4-PL curves and statistical analysis were done using GraphPad Prism software version 7 for Windows (CA, USA). Treatment effects in each donor were assessed by non-parametric one-way ANOVA analysis. BV2 data were analysed by parametric one-way ANOVA. Fisher post-hoc tests were only performed if statistical significance was achieved (*p* < 0.05).

## Results

### Phenotypic characterisation of human MSCs in vitro

**Fig. 2:**
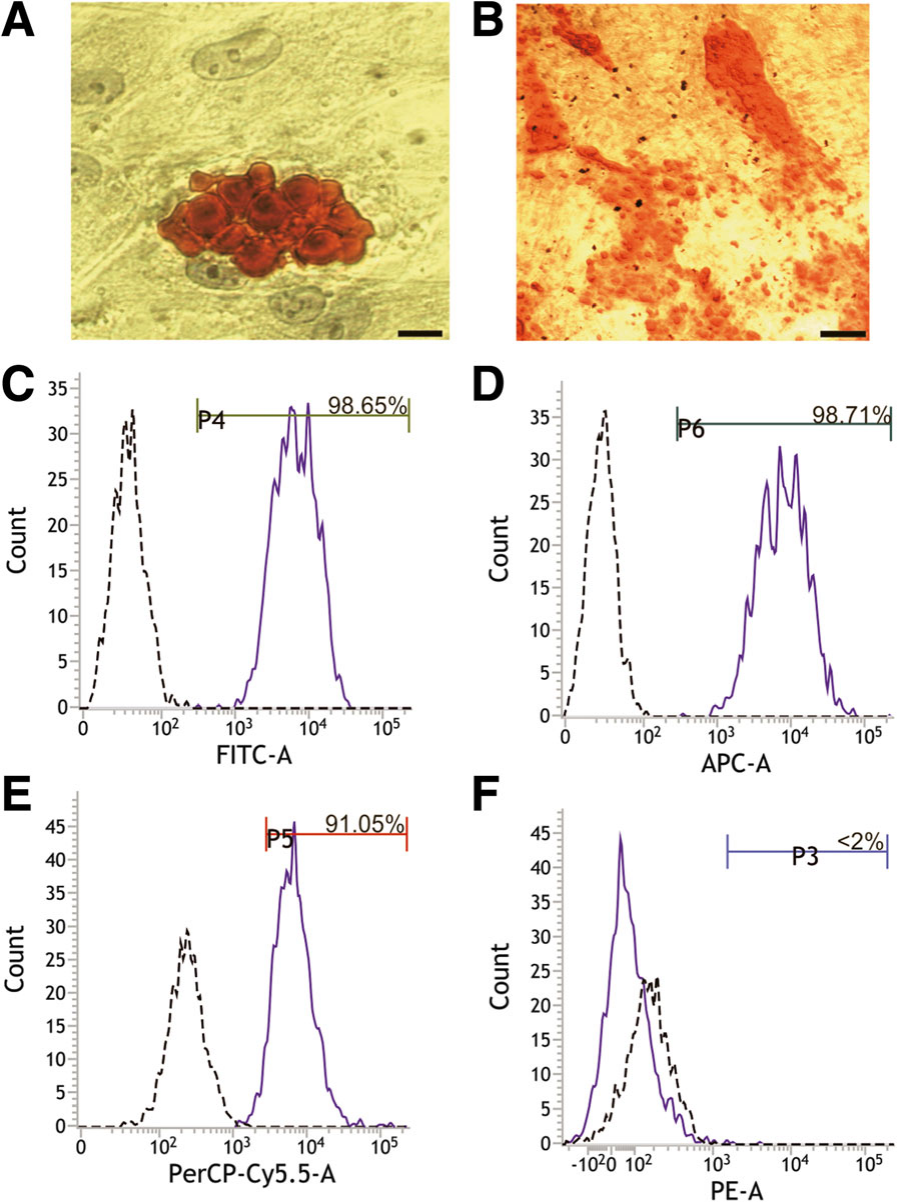
Characterisation of MSCs. Details of MSCs differentiated into adipocytes (**a**) and osteocytes (**b**). *Scale bar*: 25 μm (**a**) and 200 μm (**b**). Flow cytometry graphs showing the presence of CD90 (**c**), CD73 (**d**) and CD105 (**e**), and the absence of markers from other cell types (**f**). Percentages represent the average of all donors. Flow cytometry graphs were obtained from donor 3, and are representative of all donors

MSCs derived from three donors were differentiated successfully into adipocytes and osteocytes, evidenced by the presence of lipid droplets (stained in red, Fig. 2a) and calcium deposits (Fig. 2b), respectively. Different antibodies and corresponding isotype controls were used to assess expression of specific MSC-positive cell surface markers [29]. MSCs derived from different donors were, on average, 98.65% CD90-positive (Fig. 2c), 98.17% CD73-positive (Fig. 2d) and 91.50% CD105-positive (Fig. 2e). Less than 2% of the cells were positive for CD34, CD11b, CD45, CD19 and HLA-DR (Fig. 2f, Table 1). FACS analysis also indicated the presence of single-cell populations, with a low amount of debris, duplets and triplets, indicating a high proportion of normal and healthy cells (data not shown).

**Table 1:** Phenotyping characterisation of MSCs for all markers and all donors

	Donor 1	Donor 2	Donor 3	Mean ± SEM
CD90	95.97	100	100	98.65 ± 1.10
CD73	94.76	99.91	99.86	98.17 ± 1.39
CD105	80.24	99.48	94.79	91.50 ± 4.73
Other	3.23	2.07	0.21	1.83 ± 0.72

Quantification of marker expression from three independent cultures, expressed as the mean ± SEM

*MSC* human mesenchymal stem/stromal cell

### MSCs secrete basal levels of anti-inflammatory and neurotrophic mediators

**Fig. 3:**
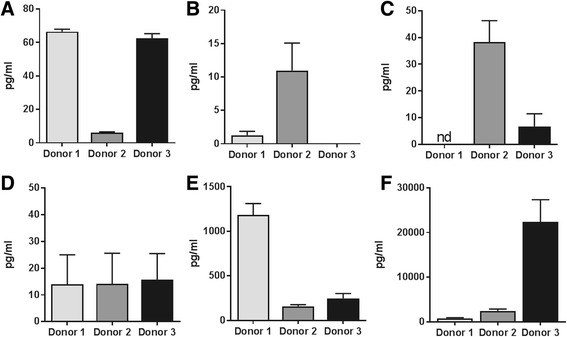
Constitutive secretion. MSCs express several anti-inflammatory cytokines and trophic factors under basal conditions (*n* = 3 experiments/donor), as measured by ELISA. Measurements of BDNF (**a**), NGF (**b**), G-CSF (**c**), IL-10 (**d**), VEGF (**e**) and IL-1Ra (**f**) in MSCs derived from the three different donors. *BDNF* brain-derived neurotrophic factor, *G-CSF* granulocyte-colony stimulating factor, *IL* interleukin, *IL-1Ra* interleukin-1 receptor antagonist, *nd* not detectable, *NGF* nerve growth factor, *VEGF* vascular endothelial growth factor

MSCs obtained from different donors were expanded and cultured, and their media were analysed for the presence of anti-inflammatory cytokines and trophic factors under basal conditions by ELISA (all values presented are expressed as mean ± SEM). MSCs constitutively expressed BDNF, IL-1Ra, NGF, VEGF, G-CSF and IL-10 (Fig. 3), although the levels secreted varied between donors; MSCs from donors 1 and 3 secreted moderate concentrations of BDNF (66.5 ± 3.6 pg/ml and 62.6 ± 4.7 pg/ml, respectively), while donor 2 only secreted 6.2 ± 0.9 pg/ml BDNF (Fig. 3a). In contrast, cells from donor 2 secreted the highest concentration of NGF (11.0 ± 7.1 pg/ml) (1.2 ± 1.0 pg/ml in donor 1 and 3.4 ± 4.0 pg/ml in donor 3; Fig. 3b).

Concentrations of G-CSF were also variable between donors, with low levels secreted in all donors (not detectable in donor 1, 38.3 ± 7.9 pg/ml in donor 2 and 6.7 ± 4.7 pg/ml in donor 3; Fig. 3c). The levels of IL-10 (Fig. 3d) were similar in all three donors (13.9 ± 11.1 pg/ml, 14.1 ± 11.5 pg/ml and 15.7 ± 9.7 pg/ml, respectively). Other factors such as VEGF were secreted in high amounts in cells from donor 1 (1182.3 ± 128.5 pg/ml); levels were lower in the other donors (donor 2, 159.3 ± 17.7 pg/ml and donor 3, 247.0 ± 55.6 pg/ml; Fig. 3e). The protein with the highest secretion in all three donors was IL-1Ra, which was in the nanogram range (0.79 ± 0.1 ng/ml in donor 1, 2.4 ± 0.4 ng/ml in donor 2), being especially high in the youngest donor (donor 3, 22.4 ± 4.9 ng/ml; Fig. 3f).

### IL-1 selectively primes MSCs to produce high levels of anti-inflammatory and pro-trophic factors

**Fig. 4:**
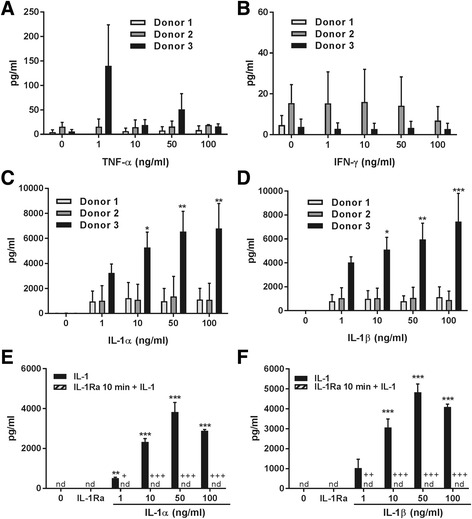
Secretion of G-CSF by MSCs. Levels of G-CSF were assayed after treating cells from different donors (*n* = 3 experiments/donor) with different concentrations of TNF-α (**a**) and IFN-γ (**b**) for 24 h, which had no significant effect on the secretion of G-CSF. Treatments with IL-1α (**c**) and IL-1β (**d**) for 24 h induced a strong increase in the secretion of G-CSF. The youngest donor presented the highest increase in secretion, as measured by ELISA (*p < 0.05, **p < 0.01, ***p < 0.001 vs untreated from same donor), despite all donors showing an increase in the secreted levels of G-CSF. The increased secretion of G-CSF in response to priming with IL-1α (**e**) and IL-1β (**f**) is completely inhibited when IL1-Ra (100 μg/ml) is added 10 min prior to the treatment. *p < 0.05, ***p < 0.001 vs untreated; ^++^ *p* < 0.05, ^+++^ *p* < 0.001 IL-1Ra condition vs cytokine treatment only. Cells from donor 3 were used in the inhibition experiment. *G-CSF* granulocyte-colony stimulating factor, *IFN-γ* interferon gamma, *IL* interleukin, *IL-1Ra* interleukin-1 receptor antagonist, *nd* not detectable, *TNF-α* tumour necrosis factor alpha

Basal concentrations of mediators were assessed in the supernatant of MSCs treated with increasing concentrations of IL-1α, IL-1β, TNF-α or IFN-γ for 24 h. Whilst TNF-α or IFN-γ had no effect on secretion of G-CSF from MSCs derived from the three donors (Fig. 4a, b), IL-1α and IL-1β induced strong G-CSF release from MSCs obtained from all of the donors (Fig. 4c, d). The magnitude of this response was different in each donor, with the highest increase observed in MSCs obtained from donor 3 (ranging from 5.9 ± 3.6 pg/ml in basal conditions to 6.8 ± 1.7 ng/ml after IL-1α and 7.4 ± 2.1 ng/ml after IL-1β). In contrast, IL-1α and IL-1β had no effect on VEGF, NGF or IL-1Ra expression (Additional file 1: Figure S1). Increased IL-10 levels were observed after IL-1α and IL-1β treatments, although this was not significant due to high variability. BDNF levels in response to IL-1α and IL-1β were highly variable with cells from the three donors showing increased, decreased or unaltered BDNF levels after IL-1α and IL-1β treatments (Additional file 1: Figure S1). Finally, release of VEGF, NGF, IL-1Ra, IL-10 and BDNF was unaltered by treatments with TNF-α or IFN-γ (Additional file 2: Figure S2, only a significant decrease in VEGF was detected in donor 1). Because cells from donor 3 produced the highest basal level of IL-1Ra and showed the most robust increase in the secretion of G-CSF in response to IL-1, all further experiments were carried out using cells from this donor.

IL-1 acts normally through IL-1 receptor type 1 (IL-1R1) [30]. To assess whether the actions of IL-1 on MSCs occurred via actions on IL-1R1, we tested the effect of human recombinant IL-1Ra (added prior to treatment with IL-1) on G-CSF release from IL-1-primed MSCs. IL-1Ra added before treatment with IL-1α or IL-1β significantly and completely blocked the priming effect of IL-1 on G-CSF secretion (*p* < 0.01–0.001; Fig. 4e, f), thus confirming that IL-1 actions were mediated by IL-1R1 activity.

### Anti-inflammatory effect of conditioned medium of MSCs on LPS-treated microglial cells is potentiated by priming of MSCs with IL-1

**Fig. 5:**
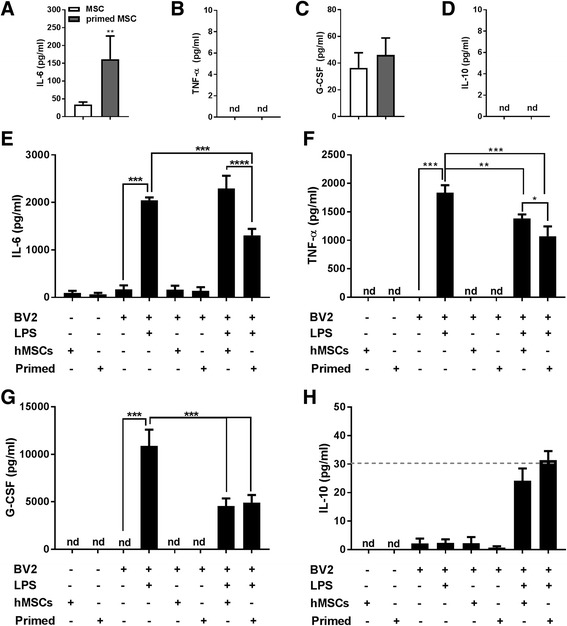
BV2 cell treatment with MSC-CM. Concentrations of human IL-6 (**a**, **p < 0.01 primed vs unprimed), TNF-α (**b**), G-CSF (**c**) and IL-10 (**d**) were measured in the CM of MSCs (*n* = 4 experiments). ELISAs to detect mouse isoforms of the same molecules (**e**, **f**, **g**, and **h**, respectively, *n* = 4 experiments) showed that the addition of MSC-CM induced significant changes in their secretion (when compared with LPS-treated BV2 cells). CM from IL-1α-primed MSCs induced a stronger response, evidenced by a larger reduction in the secretion of IL-6 (*p* < 0.001) and TNF-α (*p* < 0.05) and by an increased secretion of IL-10. Note that most IL-10 values are below the quantification limit of the assay (*dashed line*), so statistical analysis cannot be performed with these values. *p < 0.05, **p < 0.01, ***p < 0.001, ****p < 0.001. *G-CSF* granulocyte-colony stimulating factor, *hMSC* human mesenchymal stem/stromal cell, *IFN-γ* interferon gamma, *IL* interleukin, *IL-1Ra* interleukin-1 receptor antagonist, *LPS* lipopolysaccharide, *nd* not detectable, *TNF-α* tumour necrosis factor alpha

To test the hypothesis that MSCs can exert anti-inflammatory properties and could therefore be used as potent anti-inflammatory agents, we added CM from untreated or IL-1-primed MSCs to LPS-treated BV2 cells. After priming with IL-1α (see scheme in Fig. 1), MSCs exhibited increased secretion of IL-6 (*p* < 0.01; Fig. 5a) and G-CSF (Fig. 5c), whilst TNF-α and IL-10 remained undetectable (Fig. 5b, d). Stimulation of BV2 cells with LPS induced microglial activation, measured by increased secretion of IL-6 (~170 to ~2000 pg/ml, *p* < 0.001; Fig. 5e), TNF-α (~1800 pg/ml, *p* < 0.001; Fig. 5f) and G-CSF (~10,000 pg/ml, *p* < 0.001; Fig. 5g).

The addition of MSC-CM to LPS-treated BV2 microglial cells did not alter the secretion of IL-6 (Fig. 5e), but induced a significant reduction in TNF-α (~-25%, *p* < 0.01) and G-CSF (~55% reduction, *p* < 0.001), as well as a marked increase in secreted IL-10 (12-fold increase, from ~2 to ~24 pg/ml). These changes were more pronounced when LPS-treated BV2 cells were exposed to CM from IL-1α-primed MSCs, as shown by a significant reduction in IL-6 secretion (~-35%, *p* < 0.001 vs LPS, *p* < 0.0001 vs untreated CM; Fig. 5e) and in TNF-α (–41%, *p* < 0.001 vs LPS, *p* < 0.05 vs untreated CM; Fig. 5f) and a greater increase in IL-10 secretion (13-fold increase, ~30 pg/ml, vs untreated CM; Fig. 5h). The reduction in the secretion of G-CSF was unaffected by priming of MSCs with IL-1α (~55% reduction, *p* < 0.001 vs LPS treated BV2; Fig. 5g). CM from MSCs primed with TNF-α and IFN-γ were not effective at reducing the secretion of inflammatory markers (data not shown). CM (from untreated or primed MSCs) added to untreated BV2 cells had no effect on any of the mediators tested, thus discarding an unspecific effect of the addition of media from a different cell type. We measured both human and mouse isoforms of these mediators in supernatants from all conditions, and no cross-reactivity between species was observed. Finally, none of the treatments induced significant cell death or proliferation in BV2 microglial cultures (Additional file 3: Figure S3).

**Fig. 6:**
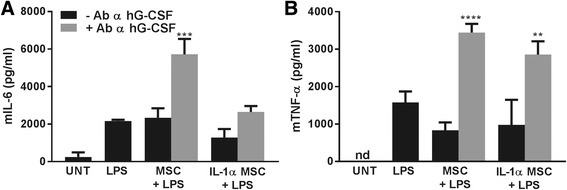
G-CSF neutralisation of MSC-CM induces raised IL-6 and TNF-α levels in BV2 cells. Addition of MSC-CM depleted of G-CSF induced higher secretion of IL-6 (**a**) and TNF-α (**b**) from LPS-stimulated BV2 cells (*p* < 0.001 for IL-6; *p* < 0.0001 and *p* < 0.01 vs non-neutralised CM, for TNF-α when CM was from unprimed or primed MSCs, respectively). *G-CSF* granulocyte-colony stimulating factor, *h* human, *MSC* mesenchymal stem/stromal cell, *IL* interleukin, *LPS* lipopolysaccharide, *nd* not detectable, *TNF-α* tumour necrosis factor alpha

Given the large amount of G-CSF secreted by MSCs in response to IL-1 preconditioning, and the fact the anti-inflammatory effect of MSC-CM was significantly enhanced by IL-1 priming, we next hypothesised that G-CSF could be a key mediator involved in these anti-inflammatory actions. To this end, we found that the levels of mouse IL-6 and TNF-α were significantly higher when human G-CSF was neutralised with a specific antibody (Fig. 6), thus confirming its role in the modulation of the secretion of these two molecules for anti-inflammatory actions on BV2 cells.

## Discussion

We describe the priming of MSCs with inflammatory cytokines to test whether this causes a secretory profile towards an anti-inflammatory and pro-trophic phenotype. We show that treatment of MSCs with IL-1α or IL-1β increased the secretion of trophic factors such as G-CSF. This was a specific effect of IL-1, because TNF-α and IFN-γ failed to induce this response.

In order to test the cells’ anti-inflammatory and pro-trophic potential, we added CM from MSCs to inflamed murine BV2 cells. Priming of MSCs induced the secretion of anti-inflammatory mediators such as IL-10 in BV2 cells, as well as a decrease in the secretion of pro-inflammatory cytokines (IL-6, TNF-α). These effects were more marked when MSCs had been primed previously with IL-1, supporting the potential use of priming treatments to induce more desirable phenotypes. We also confirmed that IL-1 exerts these effects via IL-1R1, because IL-1Ra completely blocked the increase in G-CSF after the priming. Furthermore, inhibition experiments using a specific human G-CSF neutralising antibody demonstrated that G-CSF is critical for the anti-inflammatory effect of MSCs triggered by IL-1 priming.

While first identified as a growth factor that promotes survival, proliferation and differentiation of myeloid progenitors [31], G-CSF has since been found to be a key neurotrophic factor in the CNS [32]. The G-CSF receptor is expressed throughout the adult CNS and its activation includes inhibition of apoptosis in neurons and initiation of neurogenesis in neural stem cells [33, 34].

Potentially beneficial actions of G-CSF reported in animal models include increased synaptogenesis, angiogenesis, neuroprotection, neurogenesis, plasticity and anti-apoptosis [34, 35]. In rodent models of cerebral ischaemia, G-CSF treatment has been shown to have neuroprotective effects leading to reduced infarct volume [36, 37], improved functional recovery [34, 38] and neurogenesis [39]. G-CSF has also been described as a mobilisation factor of endogenous or transplanted MSCs [40, 41], which along with all of the aforementioned features could explain some sensorimotor and functional improvements already described with MSC treatment [42, 43]. Moreover, the increased secretion of G-CSF induced by MSCs induced an M2 or M2-like functional phenotype in macrophages (which implies an anti-inflammatory and pro-regenerative phenotype) [44–46], as well as leading to higher rates of tissue remodelling and angiogenesis [47].

There is some controversy in the literature over the effects of G-CSF on experimental ischaemic stroke and brain haemorrhage [48], with positive or neutral outcomes as well as some negative results being reported [49, 50]. Clinical trials showed that the administration of G-CSF to stroke patients is safe (ClinicalTrials.gov NCT00901381, NCT00132470), but the data on efficacy have been contradictory [51] (ClinicalTrials.gov NCT009278361, NCT00132470). A study has suggested that G-CSF could be more beneficial when administered in the chronic phase to potentiate neural repair mechanisms [48]. This potential efficacy in delayed treatments favours the hypothesis that administration of cell therapies in the chronic phase leads to remodelling and repair of the injured brain. Other clinical trials assessing the safety and efficacy of G-CSF in Alzheimer’s disease (ClinicalTrials.gov NCT01617577), ALS (ClinicalTrials.gov NCT01825551), brain injury and other neurodegenerative diseases (ClinicalTrials.gov NCT02236065) are being conducted.

G-CSF may play a dual roleas it can also be produced as an autocrine protective mechanism, because neurons secrete it in ischemic conditions in an attempt to reduce neuronal apoptosis [34, 52]. In this study we have shown that MSC priming with IL-1 induces increased secretion of G-CSF (in MSCs), whilst G-CSF secretion is reduced in LPS-treated BV2 cells exposed to MSC-CM, demonstrating the dual role of G-CSF. In this case, the reduction in the secretion of G-CSF by BV2 cells might indicate that BV2 cells were responding more effectively to the inflammation.

The increase in IL-10 in BV2 cells after MSC-CM treatment adds potential to the therapeutic application of MSCs, because IL-10 is known to inactivate macrophages and to trigger matrix deposition and tissue remodelling [45, 46]. MSCs can be primed to secrete trophic factors, and they can also induce changes in the secretory phenotype of other cells, and could potentially increase the chances for injured or inflamed tissue to achieve a better recovery after an inflammatory event. In agreement with this, our data demonstrated that MSCs regulate microglial cells to secrete less inflammatory mediators and more anti-inflammatory cytokines, which may contribute to the healing and repair of the tissue.

We also need to consider the constitutive secretion of a wide variety of other cytokines. MSCs constitutively secrete high concentrations of IL-1Ra, and this is of interest because it has been shown in humans that IL-1Ra is required to be administrated repeatedly and in high doses to maintain its efficacy [53]. Therefore, MSCs from certain donors (those with a high constitutive secretion) may become a cellular system to deliver high and sustained doses of IL-1Ra. Besides, because MSCs tend to migrate to injured, inflamed or ischaemic areas [5–7], this would be a targeted delivery. This would imply a significant improvement from actual therapies, which require repeated doses of expensive recombinant human proteins [53, 54]. Other molecules that are expressed constitutively could possibly be stimulated by other priming agents not tested here.

Our results highlighted variability between MSCs from different donors, but a clear correlation between their secretome and sources (sex and age) was not found. Similarly to other studies [55, 56], we could not observe any clear relationship between the levels of these cytokines and the age or the sex of the donor. Variability between donors has been reported widely in the literature, and can be observed at the gene expression level [57, 58] and in response to differentiation stimuli [55, 58, 59]. When describing the secretome of MSCs, results are highly variable between studies, indicating once more the important variability of these cells and the existence of subpopulations [60–62]. This variability needs to be considered when designing new cell therapies because it can limit applicability.

## Conclusions

Stem cell therapies using MSCs are a promising option for the treatment of several neurological conditions because of their safety, their immunosuppressive properties and their ability to sense and reach the inflamed area, therefore potentially improving recovery and repair [1, 2, 6, 10]. In our study, we demonstrate that preconditioning treatments increased MSC secretion of anti-inflammatory mediators and trophic factors [8]. They can also trigger changes in other cells, switching them towards a more anti-inflammatory and pro-regenerative phenotype. These results highlight the possibility of modulating the secretome of MSCs, and confirm the beneficial actions they can exert when added to inflamed cells or tissues. Taken together, these results propose MSCs as an excellent candidate to be considered when designing more effective cell therapies to be used in CNS inflammatory conditions.

## Additional files

Additional file 1: Figure S1. Is showing levels of cytokines secreted by MSCs from three different donors after IL-1α or IL-1β treatment. Secretion of VEGF (**A**, **B**) and NGF (**C**, **D**) was not modified by any treatments in any of the donors, but secretion of IL-10 showed a non-significant increase in some donors (**E**, **F**). Levels of IL-1Ra were high and unchanged after IL-1 treatments (**G**, **H**). Changes in the levels of BDNF were different in each donor, showing significance in some donors (**I**, **J**) (*n* = 3 experiments/donor). *p < 0.05, **p < 0.01, ***p < 0.001 vs untreated. (TIF 461 kb)

Additional file 2: Figure S2. Is showing levels of cytokines secreted by MSCs from three different donors, after TNF-α or IFN-γ treatment. Secretion of VEGF (**A**, **B**), NGF (**C**, **D**), IL-10 (**E**, **F**), IL-1Ra (**G**, **H**) and BDNF (**I**, **J**). Donor 1 showed a significant decrease in the amount of VEGF, but donors 2 and 3 showed no response. No significant changes were detected in secretion of NGF, IL-10, IL-1Ra and BDNF (*n* = 3 experiments/donor). *p < 0.05, **p < 0.01, ***p < 0.001 vs untreated. (TIF 470 kb)

Additional file 3: Figure S3. Is showing measurement of cell death and proliferation of BV2 cells in CM treatment experiments. LDH was measured in supernatants (A) and cell lysates (B) as indirect measurements of cell death and proliferation. None of the treatments induced significant cell death or proliferation. (TIF 585 kb)

## Abbreviations

BDNF: Brain-derived neurotrophic factor
CM: Conditioned media
CNS: Central nervous system
ELISA: Enzyme-linked immunosorbent assay
IFN-γ: Interferon gamma
IL: Interleukin
IL-1R1: Interleukin-1 receptor type 1
IL-1Ra: Interleukin-1 receptor antagonist
LDH: Lactate dehydrogenase
MSC: Mesenchymal stem/stromal cell
nd: Not detectable
NGF: Nerve growth factor
SEM: Standard error mean
TNF-α: Tumour necrosis factor alpha
VEGF: Vascular endothelial growth factor
