# Supplementary figures and images for: Interleukin-1 primes human mesenchymal stem cells towards an anti-inflammatory and pro-trophic phenotype in vitro
Source: Stem Cell Res Ther. 2017 Apr 17;8:79. doi: 10.1186/s13287-017-0531-4 (PMC5393041; doi:10.1186/s13287-017-0531-4)

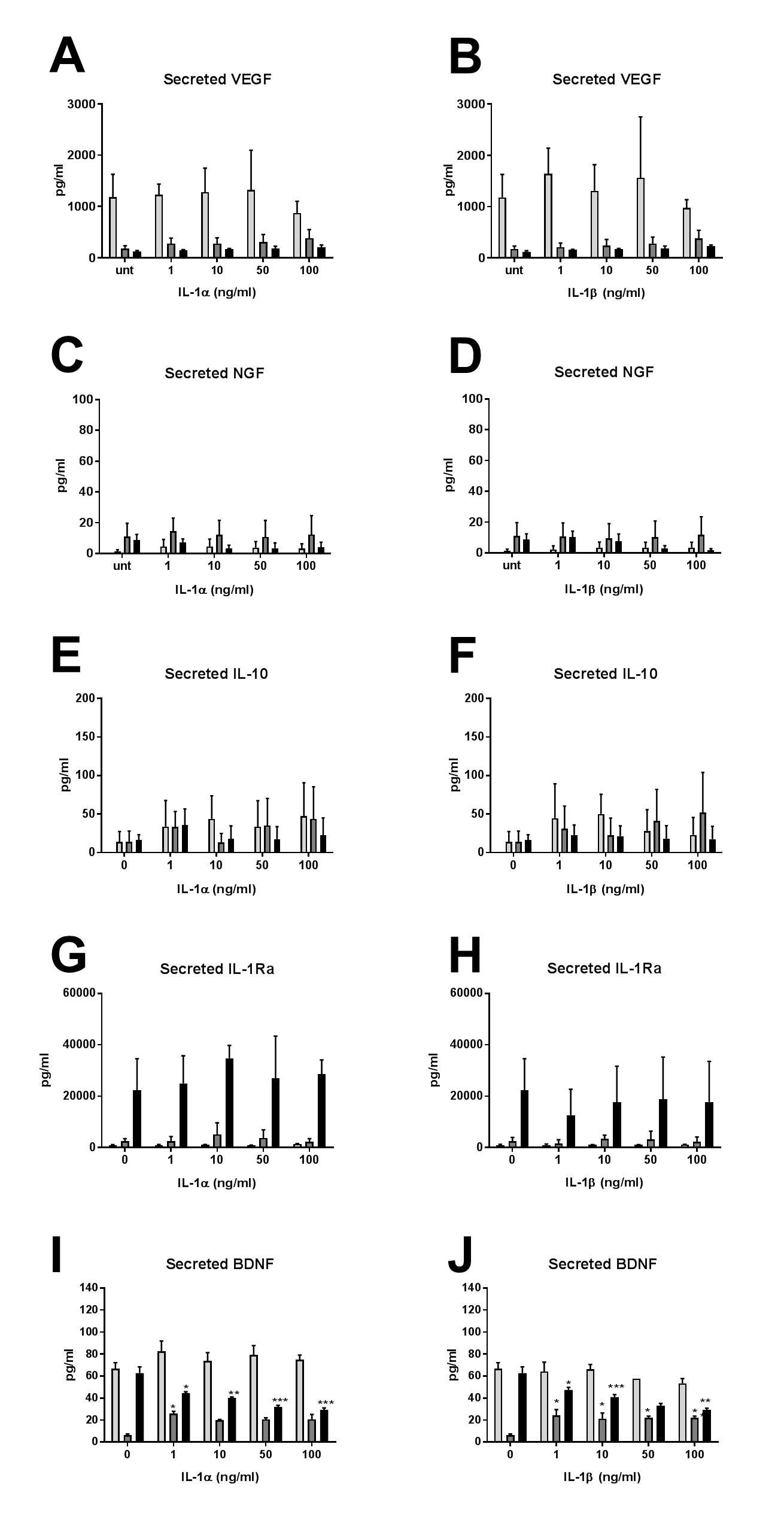

Supplement: Supplementary file 1 — Is showing levels of cytokines secreted by MSCs from three different donors after IL-1α or IL-1β treatment. Secretion of VEGF (A, B) and NGF (C, D) was not modified by any treatments in any of the donors, but secretion of IL-10 showed a non-significant increase in some donors (E, F). Levels of IL-1Ra were high and unchanged after IL-1 treatments (G, H). Changes in the levels of BDNF were different in each donor, showing significance in some donors (I, J) (n = 3 experiments/donor). *p < 0.05, **p < 0.01, ***p < 0.001 vs untreated. (TIF 461 kb) [file 13287_2017_531_MOESM1_ESM.tif]

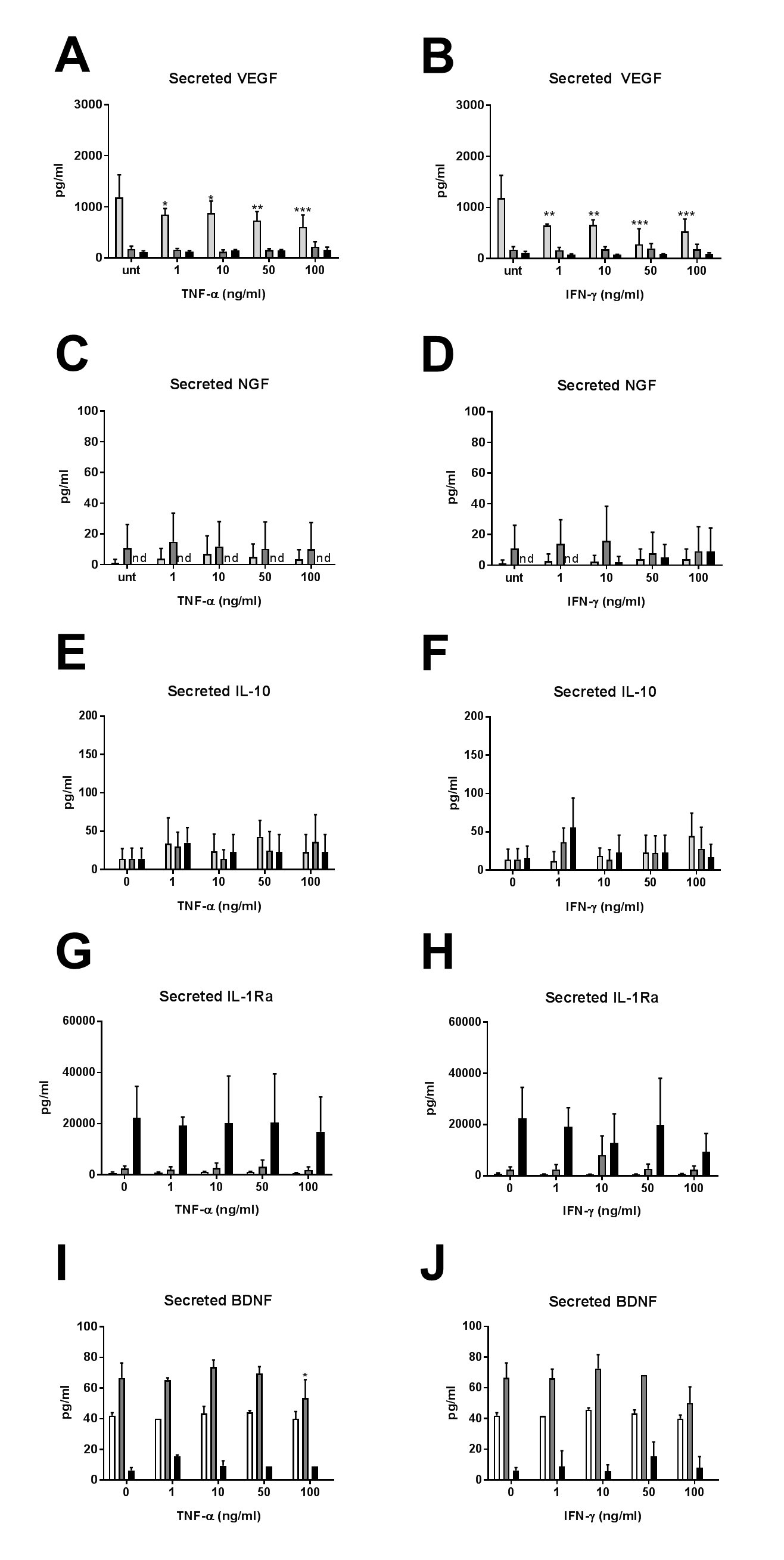

Supplement: Supplementary file 2 — Is showing levels of cytokines secreted by MSCs from three different donors, after TNF-α or IFN-γ treatment. Secretion of VEGF (A, B), NGF (C, D), IL-10 (E, F), IL-1Ra (G, H) and BDNF (I, J). Donor 1 showed a significant decrease in the amount of VEGF, but donors 2 and 3 showed no response. No significant changes were detected in secretion of NGF, IL-10, IL-1Ra and BDNF (n = 3 experiments/donor). *p < 0.05, **p < 0.01, ***p < 0.001 vs untreated. (TIF 470 kb) [file 13287_2017_531_MOESM2_ESM.tif]

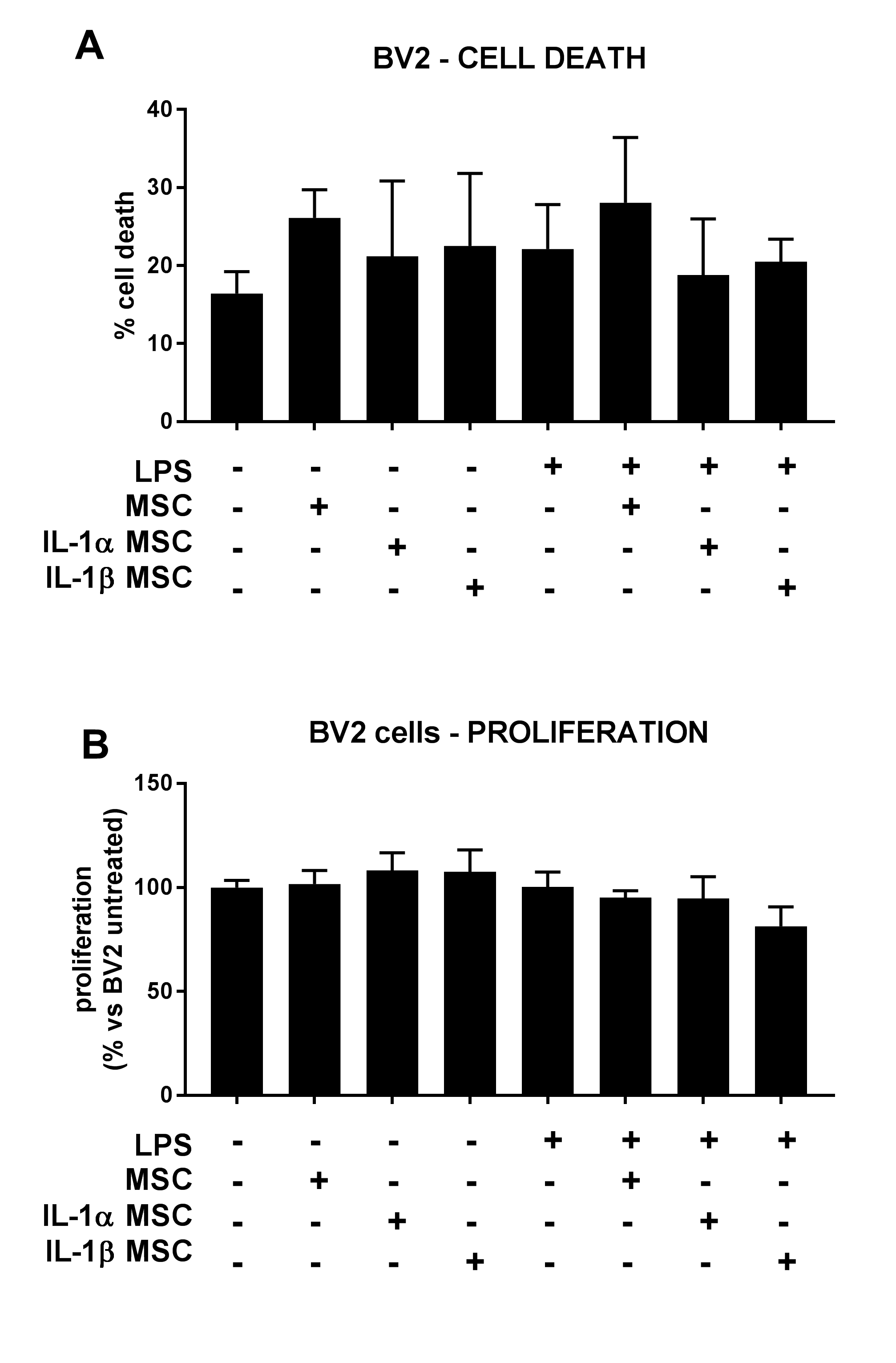

Supplement: Supplementary file 3 — Is showing measurement of cell death and proliferation of BV2 cells in CM treatment experiments. LDH was measured in supernatants (A) and cell lysates (B) as indirect measurements of cell death and proliferation. None of the treatments induced significant cell death or proliferation. (TIF 585 kb) [file 13287_2017_531_MOESM3_ESM.tif]
